# Equity impacts of cycling investment in England: A natural experimental study using longitudinally linked individual-level Census data

**DOI:** 10.1016/j.ssmph.2023.101438

**Published:** 2023-06-01

**Authors:** Richard Patterson, David Ogilvie, Anthony A. Laverty, Jenna Panter

**Affiliations:** a MRC Epidemiology Unit, University of Cambridge, Box 285 Institute of Metabolic Science, Cambridge, CB2 0QQ, UK; b Public Health Policy Evaluation Unit, Imperial College London, Reynold Building, St Dunstan's Road, London, W6 8RP, UK

**Keywords:** Cycling, Evaluation, Intervention, Natural experiment, Active travel

## Abstract

**Background:**

Cycling is beneficial for health and the environment but the evidence on the overall and differential impacts of interventions to promote cycling is limited. Here we assess the equity impacts of funding awarded to support cycling in 18 urban areas between 2005 and 2011.

**Methods:**

We used longitudinally linked 2001 and 2011 census data from 25,747 individuals in the Office for National Statistics Longitudinal Study of England and Wales. Logistic regression was used to assess the impacts of funding on commute mode as the interaction between time and area (intervention/comparison) in individual-level difference-in-difference analyses, adjusting for a range of potential confounding factors. Differential impacts were examined by age, gender, education and area-level deprivation, and uptake and maintenance of cycling were examined separately.

**Results:**

Difference-in-difference analyses showed no intervention impact on cycle commuting prevalence in the whole sample (AOR = 1.08; 95% CI 0.92, 1.26) or among men (AOR = 0.91; 95% CI 0.76, 1.10) but found an intervention effect among women (AOR = 1.56; 95% CI 1.16, 2.10). The intervention promoted uptake of cycling commuting in women (AOR = 2.13; 95% CI 1.56, 2.91) but not men (AOR = 1.19; 95% CI 0.93, 1.51). Differences in intervention effects by age, education and area-level deprivation were less consistent and more modest in magnitude.

**Conclusions:**

Living in an intervention area was associated with greater uptake of cycle commuting among women but not men. Potential gender differences in the determinants of transport mode choice should be considered in the design and evaluation of future interventions to promote cycling.

## Highlights

• Investment can increase levels of cycling and walking.
• These increases might differ between men and women.
• Funding for cycling promotion might support greater gender equality in cycling.
• This should be considered in the design and evaluation of interventions to promote cycling.

## 1 Introduction

Transport systems across the globe have contributed to people's increased dependence on motor vehicles at the expense of walking and cycling, threatening attempts to meet UN Sustainable Development Goals on climate action, sustainable cities and health. High motor vehicle use contributes to road traffic collisions and poor air quality, which are both responsible for a substantial health burden (GBD 2015 Risk Factors Collaborators, 2016; Giles-Corti et al., 2016). Reliance on cars for transport also partly explains the large number of adults who are insufficiently physically active, with an estimated 28% of adults globally failing to meet recommendations, rising to 34% of men and 42% of women in England (Guthold, Stevens, Riley, & Bull, 2018; Scholes, 2017).

Only around 3% of adults in England cycle to work as their usual mode of travel, with an additional 11% walking (Goodman, 2013). As in many countries, overall levels of cycling mask considerable area-level variation; for example, 19% of commuters in Oxford cycled in 2011 compared with 2% in Leeds. There may be a range of reasons for this including heterogeneity in the local environment, which is a key determinant of physical activity (Koohsari et al., 2015). Despite this, there is a lack of high-quality longitudinal evaluative studies on how changes in the environment could increase physical activity at a population level, and their potential differential effects (NIHR, 2018). Most existing work has evaluated the impact of single pieces of infrastructure (Brown, Werner, Tribby, Miller, & Smith, 2015) or changes in a single setting, such as a workplace (Patterson, Ogilvie, & Panter, 2020) or school (Love, Adams, & Sluijs, 2019). Recent reviews have highlighted the dearth of literature assessing interventions operating at multiple scales, including those which combine behavioural and environmental components (NIHR, 2018).

In addition to geographical variation in walking and cycling, considerable differences are seen between population groups. For example, 4.0% of commuting men in England cycle to work, compared with 2.3% of women; for walking, the proportions are 8.7% and 16.6% respectively (Patterson, Panter, et al., 2020). Those with a university degree are less likely to walk to work than those without (8.7% vs. 13.4%), with little variation seen in cycling (3.4% vs. 3.2%) (Patterson, Panter, et al., 2020). However, despite substantial heterogeneity in patterns of walking and cycling to work (collectively known as active commuting), there has been little focus on differential impacts of interventions across these groups, despite some studies finding greater intervention effects in less affluent areas (Goodman, 2013).

A review of policies to promote active travel found them to be most effective when implemented as comprehensive packages rather than individual policies (Winters, Buehler, & Götschi, 2017). Few studies have evaluated the impact of interventions that include behavioural and environmental components, particularly in relation to cycling (NIHR, 2018). One example examined the impact of town-wide funding given to 18 areas in England to get more people cycling more often using serial cross sectional census data (Goodman, Panter, Sharp, & Ogilvie, 2013). The 18 areas comprised 6 towns and cities awarded funding as Cycling Demonstration Towns (CDTs) in 2005 and a further 12 Cycling Cities and Towns (CCTs) in 2008, with local priorities dictating the exact nature of the intervention. The study found that, cycling and walking increased in intervention areas relative to a comparison group (Goodman et al., 2013). This was consistent with findings from serial cross-sectional analyses of Active People Survey data to evaluate CDTs (Cavill, Muller, Mulhall, & Harold, 2009). Building on this work, here we use within-person longitudinal repeated measures of commute mode to examine the equity impacts of these town-wide initiatives on active commuting, switching to, or from, active commute modes, and any differential effects by gender, age, education and area-level deprivation.

## 2 Methods

### 2.1 Intervention

In 2005 six CDTs were announced (Darlington, Lancaster, Exeter, Aylesbury, Derby and, Brighton and Hove) and in 2008, 12 new CCTs were announced (Blackpool, Bristol, Cambridge, Chester, Colchester, Leighton/Linslade, Shrewsbury, Southend-on-Sea, Southport with Ainsdale, Stoke, Woking and York). Together these 18 areas received additional funding to promote cycling and were considered the intervention group here. Local authorities matched central government funds, which resulted in total spending of around £135million, or approximately £16 per person per year, more than 10 times the national average at that time. Capital investment accounted for around 75% of expenditure, with the remaining 25% being revenue expenditure, such as training and events. More than 360 km of cycle lanes and tracks were laid, of which more than 215 km were not on roads, an increase of more than 30% over the study period. Measures such as traffic calming, speed restriction and improved junctions were also added in many areas. Neighbourhood events, workplace support for journey planning, and school-based cycle training (Bikeability) were also provided. Local policy makers in each town were supported by an arms-length government body (CyclingEngland, 2010) to develop and prioritise their plans, while collaboration across intervention areas was encouraged to exchange experiences and share skills (Sustrans, 2017). Although the behavioural and environmental intervention components were not restricted to commuting behaviour, workplaces were targeted by many of the intervention areas as part of their programme. More details are presented in Supplemental Tables 1 and 2, and elsewhere (Goodman et al., 2013; Sustrans, 2017). In line with UK Medical Research Council guidance, as an event outside the control of researchers we consider this intervention to be a natural experiment (Craig et al., 2011).

### 2.2 Data

The Office for National Statistics-Longitudinal Study (ONS-LS) contains census data for a 1% representative sample of the population of England and Wales, selected based on 4 dates of birth (4 of 365) (Lynch, Leib, Warren, Rogers, & Buxton, 2011; Office for National Statistics, 2019). We used linked data from 2001 to 2011, allowing individual-level changes over time to be examined. Census participation is a legal requirement, resulting in a response rate of greater than 90% (Office for National Statistics, 2015). Respondents aged at least 16 years, employed at both time-points and who lived in the same local authority area in 2001 and 2011 were included; those working from home were excluded. Missing data in census returns are imputed by the ONS using a complex and validated methodology resulting in complete data for all eligible participants (Office for National Statistics, 2012). The study met the data curator's guidelines for protection of human subjects concerning their safety and privacy.

### 2.3 Outcomes

Participants reported a single usual commute mode from a list of options. Outcomes of interest were a) cycling to work b) walking to work c) cycling or walking to work (groups a and b combined). Although funding was specifically directed at cycling, it is possible that walking might have been influenced by policies to encourage both walking and cycling (Goodman et al., 2013; Jo Christensen, Chatterjee, Marsh, Sherwin, & Jain, 2012).

### 2.4 Exposure

Assignment to intervention and comparison groups was based on local authority area of residence. Boundaries from 1991 were used for assignment as this was consistently linked with participant's home location at both time-points. There were some minor changes to the organisation of local authorities between 1991 and 2001 (and 2011), more details of which are given in Supplemental Table 3.

#### 2.4.1 Comparison groups

Multiple comparison groups were used to allow triangulation and an exploration of potential sources of bias, in line with guidance for natural experiment evaluation (Craig et al., 2011). We used four comparisons:

1. Matched comparison group, comprising participants resident in local authority areas matched to intervention areas on important characteristics, using ONS corresponding authorities (Office for National Statistics, 2010). Corresponding authorities are based on a range of domains, including age, ethnicity and the industrial sectors responsible for local employment. The three most closely matched authorities were included for each intervention area (Supplemental Tables 3 and 4).
2. Unfunded comparison group made up of participants living in areas that unsuccessfully applied for CDT or CCT funding (Supplemental Table 3).
3. Participants living in areas successful in a later 2013 cycling funding scheme (Cycle City of Ambition; CCA) in 2013 (excluding those who were included as intervention areas) (Supplemental Table 3).
4. All residents of non-intervention local authorities in England excluding London.

The matched comparison group was selected a-priori as the primary comparator, similar to previous work that showed it was most closely aligned to the intervention group in terms of the pre-existing trend in cycling (Goodman et al., 2013). However, this group is unable to capture characteristics of the area or its leadership that prompted an application for funding and to its success. The unfunded comparison group controls for factors associated with applying for funding, such as pro-cycling local leaders, a potential source of confounding by indication, but could not capture factors leading to funding success. Areas awarded funding for another later scheme in 2013 (CCA) which therefore fell outside of the study period of these analyses, aimed to control for factors associated with a successful funding bid, specifically those that are relatively time invariant. The non-London England-wide group provides a comparison with the national trend in active commuting, although it does not control for specific characteristics of intervention areas. London was excluded as the intervention was not available to London local authorities and London differs substantially from other English urban areas in terms of travel patterns.

### 2.5 Analyses

The characteristics of intervention and comparison participants were compared across a range of individual- and area-level factors measured at baseline (2001). These were age, gender, ethnicity, highest educational qualification, occupation based socio-economic group (National statistics socioeconomic classification - NSSEC), long-term illness, self-reported health, number of cars, housing tenure, working status, marital status and quintile of Carstairs index of ward of residence. Carstairs is a composite of area-level male unemployment, lack of car ownership, overcrowding, and social class of household head (Boyle, Norman, & Rees, 2004; P. Norman, Boyle, & Rees, 2005; P. Norman, 2017; P. D. Norman, 2016; P. D. Norman & Darlington-Pollock, 2017; P. Norman & Boyle, 2014). A ward is an area of England with a mean population of 6600.

A logistic model was used to estimate the odds ratio of active commuting, with analyses conducted separately for cycling, walking and cycling/walking with each comparison group. The intervention effect was estimated as the interaction between time (2001/2011) and area (intervention/comparison) in an individual-level difference-in-difference analysis, accounting for clustering of multiple observations within individuals. This estimate represents the difference between intervention and comparison areas in the difference in cycle commuting prevalence between 2001 and 2011. Analyses were conducted unadjusted and adjusted for the factors listed above.

Differential effects of the intervention were tested with a 3-way interaction between time, intervention and the modifier to test whether estimates of intervention and time differed over the modifier in adjusted analyses. We examined age (16–39 years/40+ years), gender (men/women) and two markers of socioeconomic position, namely education (degree/no degree) and area-level deprivation (quintiles 1 and 2/quintiles 4 and 5 of Carstairs Index). Stratified analyses were used to explore these differences.

The difference-in-difference approach was unable to differentiate between areas that saw increased active travel uptake and a reduction in those ceasing to commute actively. Both have implications at the population level, but it is important to distinguish the two (Panter & Ogilvie, 2017). Therefore, additional adjusted logistic regression analyses were carried for each outcome and comparison group to examine the likelihood of active commuting uptake in 2011 and the likelihood on maintaining active commuting in 2001 and 2011.

#### 2.5.1 Sensitivity analyses

Although Cambridge was in the intervention group, it was considered to be an outlier in terms of cycling prevalence and the age profile of its cyclists, therefore sensitivity analyses were conducted excluding Cambridge and its matched comparison areas. Funding was targeted at towns and cities, therefore rural areas in some intervention local authorities would not have received additional investment, despite being assigned to the intervention group. The impact of the resulting differences between some intervention areas and local authority boundaries was explored with analyses excluding participants who lived in an area with a population density of <1000 people per square kilometre. Analyses were conducted excluding participants with imputed data. Finally, analyses with additional adjustment for the presence of a child in the household and of moving home examined the potential impact of those factors (Chatterjee, Sherwin, & Jain, 2013).

## 3 Results

### 3.1 Sample characteristics

Of the 37,263 ONS-LS participants who were employed in 2001 and 2011 and lived in an intervention or matched control area in 2001, 29,354 remained in the same area in 2011. Of these, 3607 were excluded for working from home in either 2001 or 2011. This left a total of 25,747 participants, of whom 19,374 lived in a matched comparison area (Table 1). Intervention and matched comparison participants were generally similar, with differences in prevalence less than 2% except for marital status (56% and 59% were married respectively) and number of cars/vans (49% and 53% had more than one household car respectively) (Table 1). Area-level deprivation did not differ substantially in the highest or lowest quintiles, but within the middle three quintiles the intervention participants were more likely to live in more deprived areas than comparison participants. 84% of intervention participants travelled to work by motor vehicle compared with 86% of comparison participants, although larger differences existed between individual local authority areas, e.g. 14% and 17% of participants in Cambridge and York cycled to work, while in Stoke and Blackpool this was 3% (Supplemental Table 5). The characteristics of other comparison groups are presented in Supplemental Table 6.

**Table 1:** The characteristics of the intervention and matched comparison groups.

		Intervention	Comparison	p-value
		**N** = **6,373**	**N** = **19,374**	
Gender	Male	3,299 (51.8%)	10,305 (53.2%)	0.048
	Female	3,074 (48.2%)	9,069 (46.8%)	
Age (years)	16–29	1,347 (21.1%)	4,204 (21.7%)	0.58
	30–39	2,107 (33.1%)	6,472 (33.4%)	
	40–49	2,019 (31.7%)	6,055 (31.3%)	
	50+	900 (14.1%)	2,643 (13.6%)	
Ethnicity	Minority ethnicity	241 (3.8%)	703 (3.6%)	0.57
	White	6,132 (96.2%)	18,671 (96.4%)	
Highest qualification	Less than 5 GCSE A-C	2,520 (39.5%)	7,508 (38.8%)	<0.001
	5 GCSE A-C no degree	2,308 (36.2%)	7,489 (38.7%)	
	Degree	1,545 (24.2%)	4,377 (22.6%)	
Marital Status	Unmarried	2,806 (44.0%)	8,016 (41.4%)	<0.001
	Married	3,567 (56.0%)	11,358 (58.6%)	
Limiting long term illness	No illness	6,029 (94.6%)	18,416 (95.1%)	0.15
	Has illness	344 (5.4%)	958 (4.9%)	
Self-reported health	Good	5,059 (79.4%)	15,503 (80.0%)	0.51
	Fairly good	1,161 (18.2%)	3,435 (17.7%)	
	Not good	153 (2.4%)	436 (2.3%)	
Working status	Full time	4,923 (77.2%)	15,024 (77.5%)	0.62
	Part time	1,450 (22.8%)	4,350 (22.5%)	
Number of cars/vans	No car	571 (9.0%)	1,375 (7.1%)	<0.001
	One car	2,691 (42.2%)	7,673 (39.6%)	
	More than one car	3,111 (48.8%)	10,326 (53.3%)	
Housing tenure	Owner	5,347 (83.9%)	16,519 (85.3%)	0.008
	Non-owner	1,026 (16.1%)	2,855 (14.7%)	
Occupation group	Managerial	2,480 (38.9%)	7,637 (39.4%)	0.77
	Intermediate	2,119 (33.2%)	6,397 (33.0%)	
	(Semi-)routine	1,774 (27.8%)	5,340 (27.6%)	
Commute mode in 2001	Motor vehicle	5,328 (83.6%)	16,711 (86.3%)	<0.001
	Cycle	306 (4.8%)	657 (3.4%)	
	Walk	739 (11.6%)	2,006 (10.4%)	
Area-level deprivation	Least deprived	1,354 (21.3%)	4,198 (21.7%)	<0.001
	2	1,086 (17.1%)	4,913 (25.4%)	
	3	1,662 (26.1%)	4,194 (21.7%)	
	4	1,467 (23.0%)	3,904 (20.2%)	
	Most deprived	798 (12.5%)	2,133 (11.0%)	
Moved home between 2001 and 2011	Did not move home	4,150 (65.1%)	12,564 (64.8%)	0.70
	Moved home	2,223 (34.9%)	6,810 (35.2%)	
Dependent child in household	No dependent child(ren)	3,313 (52.1%)	9,480 (49.0%)	<0.001
	1+ dependent child(ren)	3,051 (47.9%)	9,881 (51.0%)	

Data are presented as n (%).

Interaction tests indicated some evidence for interaction between the intervention and gender, age, education and area-level deprivation. Subsequent analyses were presented stratified by each of these separately, even where estimates did not appear to show meaningful differences. Stratifying by more than one factor simultaneously was not possible due to sample size constraints.

### 3.2 Individual-level difference-in-difference analyses

**Fig. 1:**
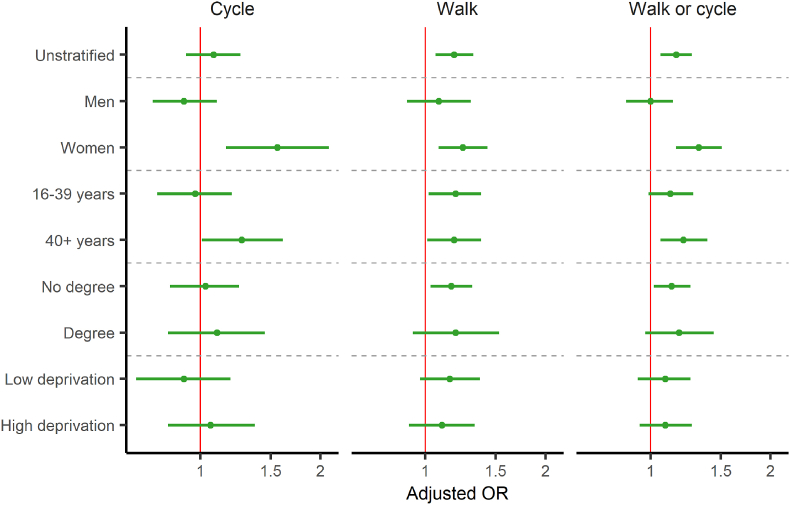
Coefficient plot of difference-in-difference adjusted odds ratio and 95% CIs across the three outcomes with the matched comparison group in unstratified and stratified analyses Source: ONS Longitudinal Study, Estimates adjusted for: age, gender, ethnicity, highest educational qualification, occupation based socio-economic group, long-term illness, self-reported health, number of cars, housing tenure, working status, marital status and quintile of Carstairs index of ward of residence (a composite measure of area-level deprivation). OR = Odds ratio; CI = Confidence interval.

Individual-level difference-in-difference analyses of cycle commuting prevalence among the whole sample did not indicate an intervention effect (adjusted odds ratio (AOR) = 1.08; 95% confidence interval (CI) 0.92 to 1.26) (Fig. 1). Gender stratified analyses found that the intervention led to increased cycling between 2001 and 2011 in women (AOR = 1.56; 95% CI 1.16 to 2.10) but not in men (AOR = 0.91; 95% CI 0.76 to 1.10) (Fig. 1).

Age stratified results indicated that the intervention led to increased cycling in older (AOR = 1.27; 95% CI 1.01 to 1.61) but not younger participants (AOR = 0.97; 95% CI 0.78 to 1.20), although overlapping confidence intervals limit confidence in a difference between the groups (Fig. 1). Stratification by education and area-level deprivation did not provide evidence to support differences in intervention effectiveness in either group (Fig. 1).

Analyses including walking were broadly consistent with those for cycling and in some cases larger changes were seen in walking than cycling. This is consistent with the hypothesis that these interventions supported walking in addition to cycling, at least in certain groups (Fig. 1). The use of other comparison groups also resulted in estimates that were consistent with those using the matched comparison group (Supplemental Fig. 1 & Supplemental Table 7).

### 3.3 Cycling maintenance and uptake

**Fig. 2:**
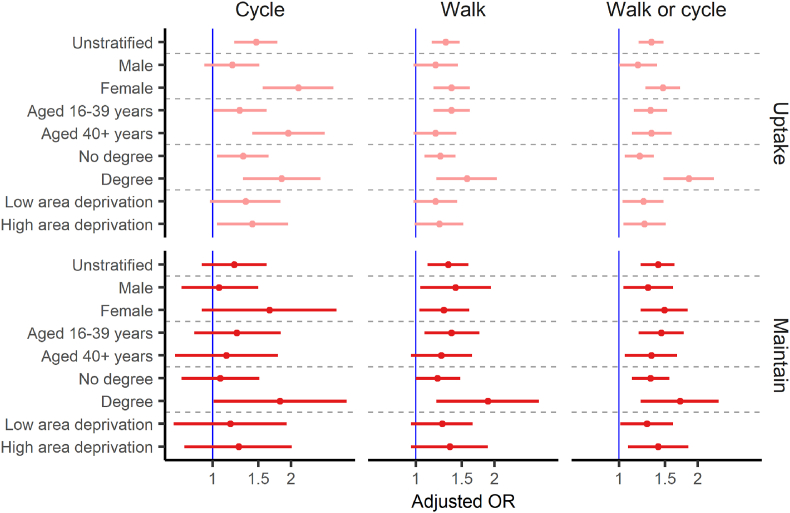
Coefficient plot of adjusted odds ratios and 95% CIs for uptake and maintaining active travel among those in intervention areas compared with those in matched areas across the three outcomes in unstratified and stratified analyses Source: ONS Longitudinal Study, Estimates adjusted for: age, gender, ethnicity, highest educational qualification, occupation based socio-economic group, long-term illness, self-reported health, number of cars, housing tenure, working status, marital status and quintile of Carstairs index of ward of residence (a composite measure of area-level deprivation). OR = Odds ratio; CI = Confidence interval.

Women in intervention areas were more likely to take up cycle commuting than those in comparison areas (AOR = 2.13; 95% CI 1.56 to 2.91) (Fig. 2 & Supplemental Table 8). This was not seen in men (AOR = 1.19; 95% CI 0.93 to 1.51). Findings for maintaining cycle commuting were inconclusive for both men and women (Fig. 2 & Supplemental Table 8).

Age and education differences were less consistent than those between men and women, but when limiting to uptake rather than maintenance of cycling, we found evidence of a positive effect in both age strata and both education strata (Fig. 2 & Supplemental Table 8). Living in an intervention area was associated with increased uptake of cycling to work among those in areas of higher deprivation (AOR = 1.42; 95% CI 1.04 to 1.95) but not in those in areas of lower deprivation (AOR = 1.34; 95% CI 0.98 to 1.82). Although the difference in these point estimates was relatively modest relative to the width of the confidence intervals (Fig. 2 & Supplemental Table 8).

Sensitivity analyses were consistent with the main analyses (Supplemental Fig. 2 & Supplemental Table 9).

## 4 Discussion

### 4.1 Summary of findings

Cycling investment in urban areas in England appeared to support an increase in the overall prevalence of cycle commuting, and its uptake, among women rather than men. In general, differences by age, education and area-level deprivation were less clear than those between men and women, although there was some evidence of effects in the higher, but not the lower stratum of area-level deprivation. Findings also suggested spill-over effects of the intervention in promoting walking.

### 4.2 Interpretation

#### 4.2.1 Gender

Relatively few intervention studies in this topic area have assessed equity impacts. This may reflect a range of practical considerations, such as sample size requirements and concerns about multiple subgroup analyses (Baker, Francis, Soares, Weightman, & Foster, 2015; Czwikla et al., 2021; Petticrew et al., 2012). Systematic reviews show inconsistent gender differences in intervention effectiveness, and those are hindered by the relative lack of primary studies (Attwood, Sluijs, & Sutton, 2016; Baker et al., 2015; Czwikla et al., 2021; Humphreys & Ogilvie, 2013; Lehne & Bolte, 2017). Gender differences in the role of the workplace environment in determining commute mode have previously been reported, with evidence that the physical environment (e.g. lockers and showers) is associated with active commuting in men, while the social environment is more important in women (Kaczynski, Bopp, & Wittman, 2010; Patterson, Ogilvie, & Panter, 2020). A 2020 systematic review suggested that safety might have greater impacts in women and recreational facilities in men (Tcymbal et al., 2020). Women have demonstrated greater perceived risk while cycling, greater concern about road safety risks and a greater preference for cycling infrastructure that separates them from other road users than men (Aldred, Elliott, Woodcock, & Goodman, 2017; Cordellieri et al., 2016; Garrard, Rose, & Lo, 2008; Prati et al., 2019). The interventions evaluated in our study varied depending on local priorities, but the majority added advanced stop lines at junctions for cyclists and provided substantial amounts of cycle lane/track (Supplemental Table 1). Combined with the events and campaigns that were held in all intervention areas (Supplemental Table 2), this might have contributed to improved perceptions of safety and had a disproportionate appeal to women. Some intervention areas had intervention components specifically aimed at women that may also explain some of the gender differences seen.

Evidence suggests that gender differences in cycling are lower in areas with higher cycling prevalence Grudgings, Hughes, and Hagen-Zanker (2021), although others found increased cycling has failed to improve the gender balance (Aldred, Woodcock, & Goodman, 2016). This raises the possibility that some of our gender differences might be partly due to relatively high levels of cycling in intervention areas (most of which had a prevalence above the national average of 3.1%) (Supplemental Table 5). Increased cycling in England has previously been linked with no change in the gender balance. We conducted sensitivity analyses excluding Cambridge as a high-cycling outlier and found this had little impact. In England and more generally, men are on average more physically active than women (Guthold et al., 2018) and cycle more (Goel et al., 2022). This reflects wider differences in travel patterns and caring responsibilities and makes understanding gendered intervention effectiveness important to ensure that inequalities are understood and addressed rather than entrenched (Hanson, 2010).

#### 4.2.2 Socio-economic position

Socio-economic differences in intervention effectiveness are less frequently examined than gender and the picture is further complicated by the use of different socio-economic markers (e.g. wealth or education; individual or area-level) (Attwood et al., 2016; Baker et al., 2015; Humphreys & Ogilvie, 2013). There is currently little evidence for socio-economic differences (Attwood et al., 2016; Czwikla et al., 2021; Humphreys & Ogilvie, 2013); however, previous analyses of CDT/CCT found they were more effective in areas of greater deprivation than less deprived areas (Goodman et al., 2013) and our findings are consistent with those, despite the different approaches used. This might reflect the fact that some intervention areas specifically targeted low-income families with certain intervention components. We also found consistently higher point estimates for those with a degree than those without but those in routine and manual occupations were more likely to cycle than those in managerial and professional occupations. Together, this suggests a complicated relationship between cycling and socio-economic position.

#### 4.2.3 Age

Differential intervention effectiveness by age is also less frequently examined than for gender, with little consistent evidence currently supporting age differences, which is in line with our findings (Attwood et al., 2016; Baker et al., 2015; Humphreys & Ogilvie, 2013). It is somewhat reassuring that the intervention appeared to be at least as effective in those aged 40 years or more as in younger adults. Physical activity generally reduces with age, despite the particular importance of being active in later life (Paterson & Warburton, 2010; Woodcock, Tainio, Cheshire, O’Brien, & Goodman, 2014). Cycling interventions that are equitable across age groups could contribute to healthy aging, which is particularly important in areas with an aging population.

#### 4.2.4 Spill-over effects

We found evidence to support beneficial spill-over effects on walking, in addition to intended impacts on cycling. This is consistent with previous findings and might reflect speed restrictions, traffic calming and improved junctions being likely to benefit pedestrians as well as those cycling (Goodman et al., 2013; Jo Christensen et al., 2012). Several intervention areas combined components supporting cycling with others restricting car use, e.g., car free days (Supplemental Table 2), in what has been labelled a carrot and stick approach (Xiao, Sluijs, Ogilvie, Patterson, & Panter, 2022). Restrictions on car use might be at least partly responsible for the changes in walking found in these analyses, where some of those discouraged from driving to work opted to walk instead.

### 4.3 Strengths and limitations

Strengths include the large representative longitudinal sample of individual-level data that is representative of the working population of England. This allowed us to examine uptake and maintenance, while reducing the impact of neighbourhood selection effects, i.e., people who like cycling moving to areas where cycling investment has created a supportive environment. The related issue of intervention areas being those that have pro-cycling leaders and/or populations cannot entirely be controlled for, although we used multiple comparison groups based on the characteristics of the intervention areas, in line with best practice (Craig et al., 2011).

This study was susceptible to bias resulting from intervention allocation that was not “as-if random” and, despite our best efforts to make controlled comparisons based on the available data, this limits our ability to infer causality (Dunning, 2012). Intervention allocation was also not coterminous with the local authority areas used to identify participants, and although our sensitivity analyses were consistent with the main results there is likely to have been some residual measurement error in exposure assignment. In addition, we used 1991 local authority boundaries as these were consistently applied to participants in both 2001 and 2011, but minor changes in local authority boundaries occurred between 1991 and 2001.

The data used comprise a cohort constructed from routinely collected administrative data, of which we used two time points. The 10-year gap between data collections has drawbacks for attributing causality as confounding events might also have occurred within the period of observation. However, the long-term nature of the dataset allows interventions that take place over several years, such as the CDT and CCTs, to be studied. It was not possible to include all potentially important covariates in analysis, either because they are not captured in census data (such as wealth and overall physical activity) or because of a high level of missingness (distance to work). Our data show that baseline cycling levels varied considerably between sites, and previous analyses showed substantial heterogeneity between intervention sites in intervention effectiveness (Goodman et al., 2013). This might reflect the fact that the intervention evaluated here was a collection of complex intervention programmes tailored to the specific needs of each area, with local content and context likely to be critical to intervention effectiveness. Unpacking the relative importance of the constituent intervention parts and local heterogeneity was not possible here due to a combination of low numbers of participants at each site and limitations of the available data.”

## 5 Conclusions

Living in a CDT/CCT area, compared with a comparison area, was associated with a greater increase in cycling during the intervention period among women but not men. These differences are potentially important due to extant lower cycling in women than men in England and elsewhere, in addition to lower physical activity more generally. Differences by age and education were less clear, but there was evidence that the intervention promoted cycling uptake in both strata of age and education. Funding for cycling promotion might provide support for greater gender equality in cycling prevalence, while potential differences between men and women should be considered in the design and evaluation of future interventions to promote cycling.

## Funding sources

David Ogilvie, Jenna Panter and Richard Patterson are supported by the 10.13039/501100000265Medical Research Council (Unit Programme number MC_UU_00006/7). Anthony A Laverty is supported by the 10.13039/501100012349NIHR School for Public Health Research (PD-SPH-2015).

## Contributors

All authors contributed to the design of the study. RP conducted the analyses and wrote the first draft of the report. JP, DO and AAL revised the report for important intellectual content.

## Ethical

External independent ethical approval was not required for this study as it used routinely collected administrative data. However, it was subjected to ethical review by the curators of the data (the UK Office for National Statistics – ONS).

## CRediT authorship contribution statement

**Richard Patterson:** Conceptualization, Methodology, Formal analysis, Writing – original draft, Writing – review & editing. **David Ogilvie:** Conceptualization, Methodology, Writing – review & editing, Funding acquisition. **Anthony A. Laverty:** Conceptualization, Methodology, Writing – review & editing. **Jenna Panter:** Conceptualization, Methodology, Writing – review & editing, Funding acquisition.

## Declaration of competing interest

We declare no competing interests.

## Data Availability

The authors do not have permission to share data.
