# Supplementary material for: Equity impacts of cycling investment in England: A natural experimental study using longitudinally linked individual-level Census data
Source: SSM Popul Health. 2023 Jun 1;23:101438. doi: 10.1016/j.ssmph.2023.101438 (PMC10251149; doi:10.1016/j.ssmph.2023.101438)
Supplement: Multimedia component 1 [file mmc1.docx]

# Supplemental material

**Supplemental Figures**

[Supplemental Figure 1: Coefficient plot of difference-in-difference estimates across the three outcomes and four comparison groups in the unstratified and stratified analyses 2](#_Toc117243307)

[Supplemental Figure 2: Coefficient plot of sensitivity analyses for difference-in-difference estimates across the three outcomes in the unstratified and stratified analyses 3](#_Toc117243308)

**Supplemental Tables**

[Supplemental Table 1: Summary of funding and certain core types of infrastructure investment across the 18 intervention towns/cities 4](#_Toc117243309)

[Supplemental Table 2: Overview of selected neighbourhood or town-wide initiatives in each town, between 2008 and 2011 5](#_Toc117243310)

[Supplemental Table 3: 1991 local authority areas used to identify participants for the intervention and the matched, unfunded and Cycle Cities of Ambition comparison groups 8](#_Toc117243311)

[Supplemental Table 4: Matched comparison group 2001 local authorities 11](#_Toc117243312)

[Supplemental Table 5: Walking and cycling to work in 2001 for each intervention area 12](#_Toc117243313)

[Supplemental Table 6: The characteristics of the intervention and different comparison groups 13](#_Toc117243314)

[Supplemental Table 7: DID estimates across the three outcomes and four comparison groups in the unstratified and stratified analyses 14](#_Toc117243315)

[Supplemental Table 8: Association between living in an intervention area and taking up cycling and maintaining cycling in 2011 compared with the matched comparison group 15](#_Toc117243316)

[Supplemental Table 9: DID effect estimates for sensitivity analyses for each outcome and strata 16](#_Toc117243317)

Supplemental Figure 1: Coefficient plot of difference-in-difference adjusted odds ratios and 95% CIs across the three outcomes and four comparison groups in the unstratified and stratified analyses


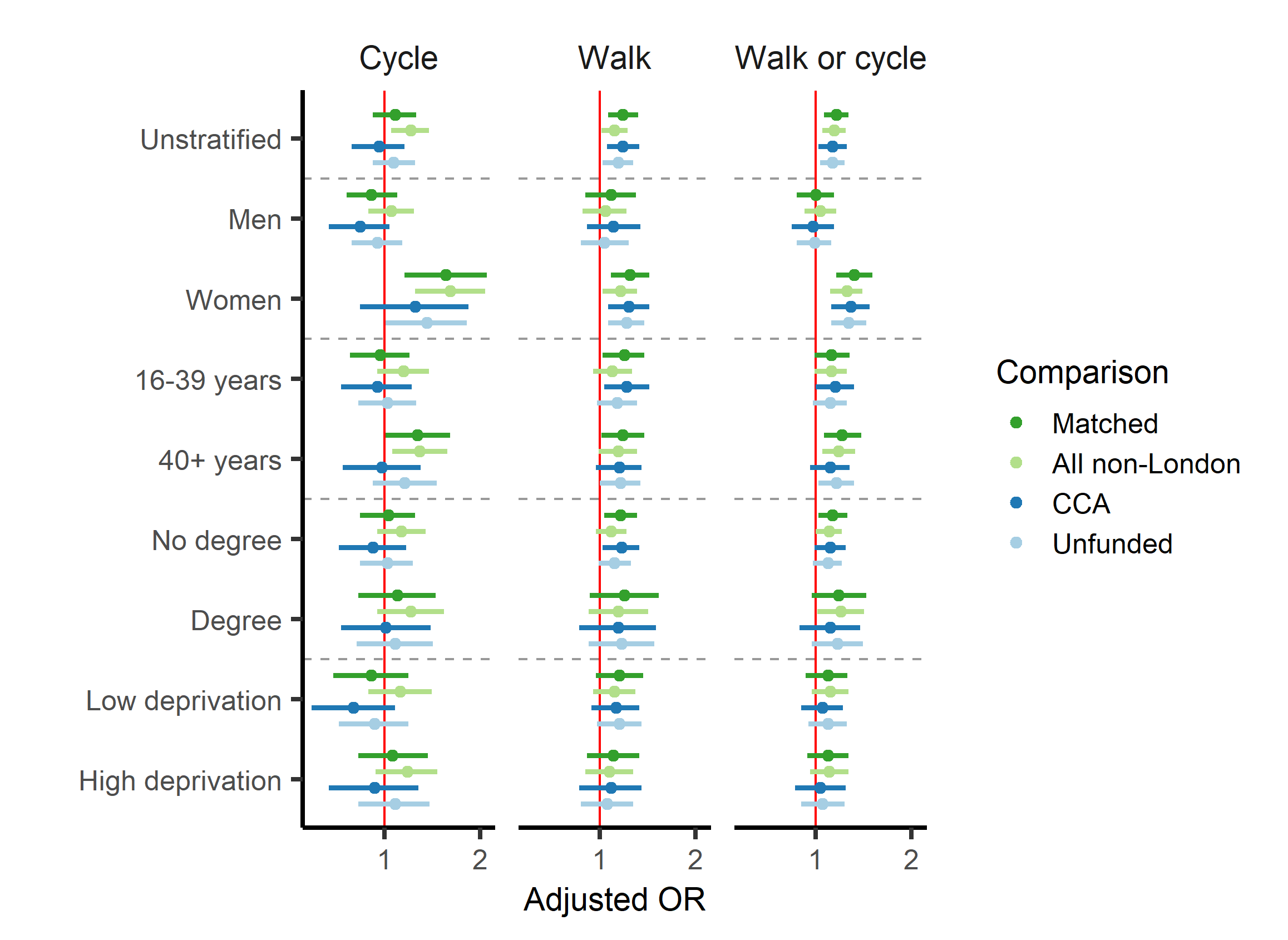


Estimates adjusted for: age, gender, ethnicity, highest educational qualification, occupation based socio-economic group, long-term illness, self-reported health, number of cars, housing tenure, working status, marital status and quintile of Carstairs index of ward of residence (a composite measure of area-level deprivation.

Comparison groups: Matched = comparison group matched using Office for National Statistics Corresponding Authorities; All non-London = all participant resident in England outside of Greater London; CCA = Areas successful in apply for funding through the Cycling Cities of Ambition funding scheme; Reject: Areas that applied for CDT or CCT funding but were unsuccessful.

OR = Odd ratio; CI = confidence interval

Source: ONS Longitudinal Study

Supplemental Figure 2: Coefficient plot of sensitivity analyses for difference-in-difference adjusted odds ratio and 95% CIs across the three outcomes in the unstratified and stratified analyses


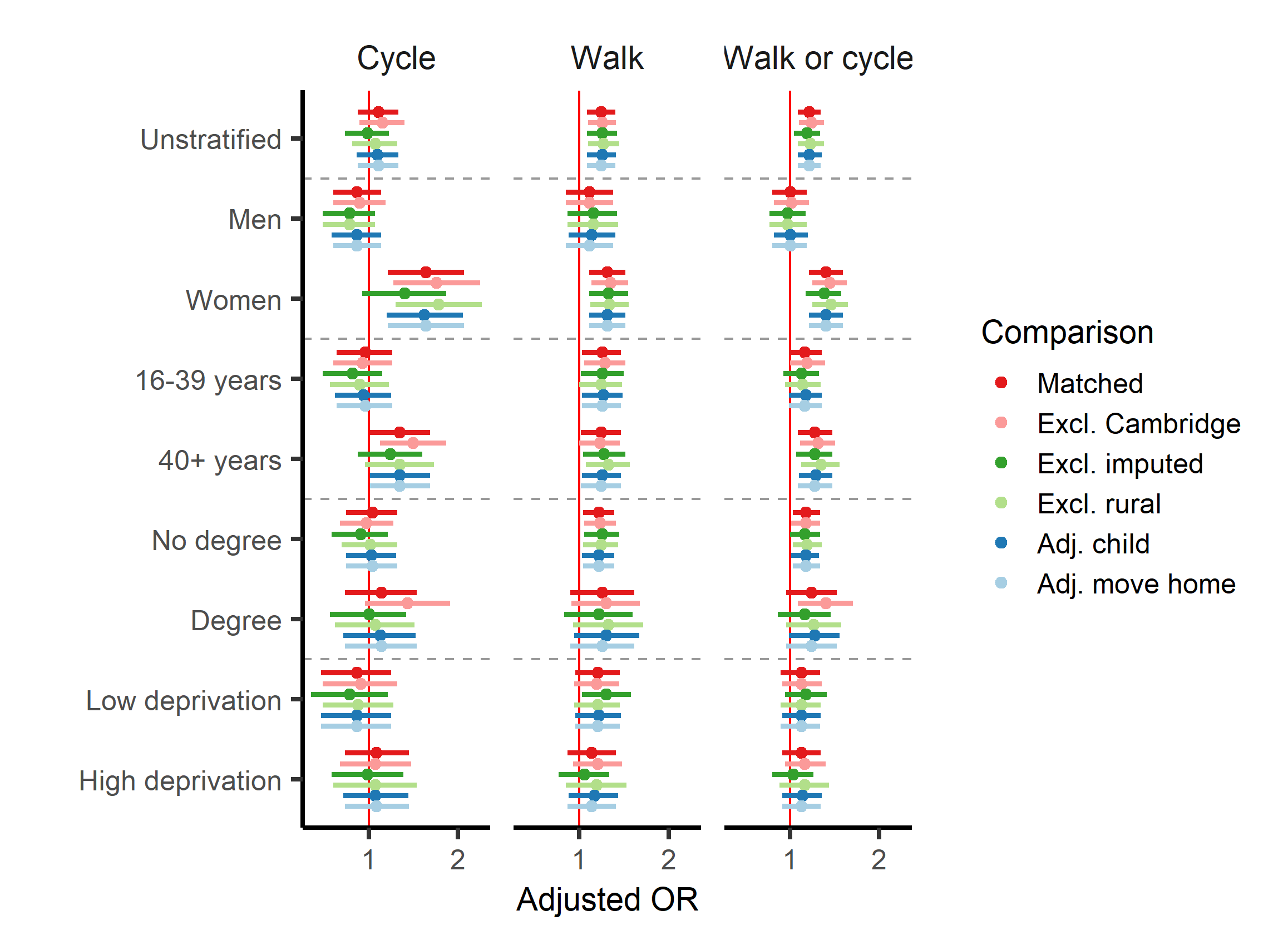


Estimates adjusted for: age, gender, ethnicity, highest educational qualification, occupation based socio-economic group, long-term illness, self-reported health, number of cars, housing tenure, working status, marital status and quintile of Carstairs index of ward of residence (a composite measure of area-level deprivation.

Comparisons: Matched = comparison group matched using Office for National Statistics Corresponding Authorities (main analyses); Excl. Cambridge = main analyses excluding Cambridge and its matched control areas; Excl. imputed = main analyses excluding participants with imputed values for any variables of interest; Excl. rural = main analyses excluding those living in areas with a population density < 1000 people per square kilometre; Adj. child = additional adjustment for the presence of at least one dependent child in the household; Adj. move home = additional adjustment for moving home within the study period.

OR = Odd ratio; CI = confidence interval

Source: ONS Longitudinal Study

Supplemental Table 1: Summary of funding and certain core types of infrastructure investment across the 18 intervention towns/cities

|  |  |  |  |  | | Cycle lanes and paths | | | |  | Cycle parking | |
| --- | --- | --- | --- | --- | --- | --- | --- | --- | --- | --- | --- | --- |
| Town name | Commuter population* | Total spend (£) | % spend on capital investment | Annual spend per head of population (£) | | On-road km | Off-road km | Total km | % increase† | Advanced stop lines | No. stands added | % increase‡ |
| Darlington | 40,572 | 5,963,339 | 80% | 11 | § | 0.7 | 10.3 | 11 | 44% | 19 | 826 | 65% |
| Derby‡‡ | 112,876 | 9,962,989 | 77% | 17 | § | 10.1 | 8.3 | 18.4 | 14% | 16 | 484 | 24% |
| Brighton & Hove†† | 110,975 | 6,237,380 | 65% | 12 | § | 6.5 | 8.3 | 14.8 | 105% | 21 | 1916 | 240% |
| Aylesbury | 37,662 | 5,707,612 | 76% | 16 | § | 4.3 | 3.7 | 8 | 35% | 0 | 90 | 36% |
| Exeter | 52,605 | 18,179,738 | 92% | 29 | § | 1.2 | 11.3 | 12.5 | 15% | 3 | 1541 | n/r |
| Lancaster with Morecambe | 42,737 | 6,814,379 | 81% | 13 | § | 5.4 | 5.0 | 10.4 | 11% | 24 | 1176 | 51% |
| York | 73,583 | 7,553,029 | 82% | 12 | ** | 10.0 ‘mostly on-road’ | | 10 | n/r | ’Built as standard’ | >450 | n/r |
| Cambridge | 85,426 | 8,954,000 | 87% | 17 | ** | 12.8 | 40.8 | 53.6 | 21% | 0 | 1944 | 23% |
| Colchester | 56,244 | 4,871,801 | 74% | 16 | ** | 0.3 | 11.9 | 12.2 | 33% | 0 | 1208 | 30% |
| Southend-on-Sea | 77,597 | 6,699,560 | 74% | 14 | ** | 3.0 | 12.0 | 15 | 88% | 21 | 2690 | 374% |
| Leighton Buzzard | 18,955 | 2,666,028 | 70% | 23 | ** | 1.2 | 2.8 | 4 | 53% | 7 | 780 | 260% |
| Woking | 54,848 | 4,341,592 | 80% | 16 | ** | 0.0 | 26.3 | 26.3 | 60% | 3 | 1269 | 74% |
| Bristol | 228,109 | 19,713,922 | 57% | 12 | ** | 18.0 | 35.1 | 53.1 | [n/r] | ’Built as standard’ | 8709 | 217% |
| Shrewsbury | 36,766 | 3,643,118 | 78% | 16 | ** | 0.7 | 5.3 | 6 | 17% | 9 | 658 | 44% |
| Stoke-on-Trent | 90,654 | 8,531,693 | 71% | 12 | ** | 0.0 | 10.8 | 10.8 | 9% | 69 | 1098 | 125% |
| Chester | 40,648 | 3,952,634 | 68% | 11 | ** | 17.0 | 53.0 | 70 | 70% | 6 | 1478 | 77% |
| Southport & Ainsdale | 38,988 | 3,669,911 | 68% | 14 | ** | 6.6 | 9.8 | 16.4 | 63% | 4 | 942 | 142% |
| Blackpool | 67,092 | 8,220,000 | 84% | 19 | ** | 0.5 | 9.8 | 10.3 | 25% | 25 | 636 | 107% |
| Total | 1,266,337 | 135,682,725 |  |  |  | 60.1 | 217.6 | 362.8 |  |  | 21412 |  |
| Mean |  |  | 72% | 15 |  |  |  |  | 37% |  |  | 123% |

Adapted from Goodman et al (1) Data extracted from the standardised end-of-programme reporting spreadsheets where available, or from narrative text where not.

n/r = not reported

* Number of commuters in the town in the 2011 census

† Percent increase in total length of cycle facilities (both on- and off-road) relative to 2008. On-road facilities included contra-flow cycle lanes (allowing bicycles to travel in both directions along a street that is one-way for motor vehicles), advanced stop lines (dedicated space for bicycle at the front of a queue for traffic lights, often accompanied by feeder routes into the advanced stop space) and mandatory cycle lanes (on-road cycle lanes indicated by a solid painted line which motor vehicles are not allowed to cross). Off-road facilities included fully-segregated cycle routes next a road or over green space, and shared use facilities for both cyclists and pedestrians.

‡ Percent increase in total number of cycle stands relative to 2008.

§ calculated as the sum of expenditure divided by the population divided by five and a half

** Calculated as the sum of expenditure divided by the population divided by three

†† Brighton and Hove focused their programme in the western part of the town, with a target population of 95,000 of the total population of 254,000 (Cycling England (2010) ‘Cycling City and Towns Project Overview: March 2010’, Cycling England)

‡‡ Derby focused their programme on children and young people, with a target population of 105,000 of the total population of 245,000 (Cycling England (2010) ‘Cycling City and Towns Project Overview: March 2010’, Cycling England)

Supplemental Table 2: Overview of selected neighbourhood or town-wide initiatives in each town, between 2008 and 2011

| **Town name** | **Major initiatives and events** |
| --- | --- |
| Darlington | - Annual cycle festival, attended by an estimated total of 5600 people across the 3 years. - Guided cycle rides attracting 160 people, and ‘tourist’ rides attracting over 150. - Cycle talks and film showings attracting 110 people. - Events around the ‘stage start’ for the 2008 and 2009 ‘Tour of Britain’ (a professional cycle race modelled on the Tour de France), attracting 8000 spectators. - Cycle training for adults and a bike loan scheme benefitting over 50 people. |
| Derby | - ‘Big Cycle Day Out’: an annual celebration of cycling in a central city park, with live music, information stalls and hands on activities. Attended by 4000-6000 people per year. There have also been cycling activities at a local annual fair (‘Chaddesden Big One’), providing exposure to 20,000 people. - BMX track events in a local park, with 2 national events and 4 regional races. The first national event attracted 6000 spectators and over 500 riders. The BMX club has a membership of over 60 families and hosts two club nights per week. - ‘The 5 Parks Ride’ a charity event across 5 of Derby’s parks which provides an easy ride for all ages. Around 750 people have taken part. - Annual ‘Bike to School Challenge’ has grown from a week to a month, pitting local schools against each other. In 2010 over 17,000 cycling journeys were recorded. Schools are also given resources to run their own events at any time of year. |
| Brighton & Hove | - ‘Annual ‘Bike Week’: bike rides and picnics, cycle maintenance workshops and cycle training, often organised by local people and organisations via grants from the programme. - Annual ‘Car Free Day’, reaching 8000-10000 in 2008/09 and 2009/10. - Cycle safety events: for example, from 2009 onwards the ‘Brighten up Brighton’ used a mixture of give-aways and enforcement to remind people about Highway Code rules on bicycle lighting. - ‘She Rides’ - women and girls’ cycle rides and maintenance, used by 120 women. - A bike loan scheme of 30 bicycles for children from low-income families. |
| Aylesbury | - Cycle festival attended by an estimated 1500 people in 2010, and coinciding with launch of a cycle map for kids. - 2 route openings in 2009 and 2010, attended by an estimated 600 people in total. - ‘Bike swap’ events, attended by around 50 people per year. - Cycle breakfasts and Dr Bike (cycle maintenance/repair) sessions attended by around 100 people per year. - Stands at local fairs and at train stations: an estimated 23,500 people exposed to the ‘Cycle Aylesbury’ campaign in total. - A website, e-bulletin and sustained radio campaign. |
| Exeter | - National Bike Week / Devon Car Free Day events: similar to Brighton (see above). - Supporting larger events: for example, in 2009 and 2010 the Cycle Exeter project hosted part of the Tour Series professional bike race, attracting up to 12,000 spectators. - Events providing skills or training (e.g. puncture repair sessions, bike shop staff training, route planning clinics). These were advertised in various ways, for example bike shops in Exeter were provided with “free training with this bike” laminated tags to attach to bikes for sale at Christmas time. - Specific campaign events - e.g. tolerance on shared use paths, ‘brighten up in winter’, women only events and campaigns. |
| Lancaster with Morecambe | - General awareness raising events included Party on the Prom (annually from 2007), Friday city centre roadshows, annual bike week activities, and a bike film festival. Approximately 25,000 people estimated to have come into contact with these events. - ‘Women on Wheels’ rides: 25 rides attended by a total of 252 women. - Bus advertising, website, e-bulletin. - Cycle rote guides: e.g. ‘Family cycling guide’ and ‘Cycling for all 2 route guide’. - 429 people have attended a free bike maintenance course (including 124 at women only courses). There have also been 78 general skills sessions for adults and 44 women only cycle skills sessions. |
| York | - Two festivals of cycling; Bike Week events; and ‘Just Try It!’ and ‘Light up your Bike’ campaigns. These were complemented by neighbourhood specific events such as bike tryout roadshows; guided cycle rides; and bike maintenance. - Newsletters delivered periodically to every household, including updated copies of York’s cycle map. - Loan scheme developed for people to buy new cycles from local bike shops at a discount. - ‘Bikes Not Barriers’ scheme to improve cycling provision for people with disabilities and learning difficulties. - ‘Fit As A Fiddle’ project aiming to encourage physical activity among people over 50. |
| Cambridge | - ‘Dust off your bike’ and ‘Cycling fun day’ organised as cycle-specific events, plus an annual ‘Bike Week’ which included a wide range of events and activities. The ‘Dust off your bike’ campaign aimed at encouraging cycling among those who had tried it in the past, and included repairing and maintaining bikes to make them road-ready. There were also stands at events organised by others, e.g. the Town and Country fair. In total, an estimated 13,000 people came in contact with the campaign. - Personalised travel planning programme in selected neighbourhoods. - Free newspaper called ‘Get Pedalling’, bus advertising, social media campaign. |
| Colchester | - 25 communities, groups and organisations received grants of up to £2000 each for projects aiming to promote cycling, as part of an open competition. - ‘Share the space’ campaign aiming to reduce conflict between cyclists and pedestrians on shared paths. - Attended a diverse range of events (e.g. music festivals and family days) to promote awareness. In doing so, the team encouraged people to make personal pledges to cycle more. In 2009 the team collected around 600 pledges and in 2010 they collected over 1000. - Cycle-specific events, e.g. launches for the improved routes and regular Dr Bike sessions during the summer. - Personalised travel planning in selected neighbourhoods. |
| Southend-on-Sea | - Group cycle rides, charity cycle rides and bikers breakfasts. - Specialist ‘Bike Week’ events, plus a presence at multiple community events. For example, the ‘Big Charge’ was a project to charge a large battery using pedal power that in turn powered that main stage at the festival. This was done through involving various community groups, including Yacht clubs, Disabled groups, and a Caribbean church group. - Bespoke marketing plan and brand, including through a website and social media. - Establishing a ‘ReCycle Centre’ with sales of recycled bikes currently totalling over 600 bikes. The council now also has a set of pool bikes available to all staff. |
| Leighton Buzzard | - A broad programme of promotional activities focused on commuters, and especially those using the stations. This included encouraging cycling to the station among current car users and rewarding people who currently cycle to the station with vouchers for a free drink. These were complemented by the ‘Get on track’ station travel plan, which tried to encourage sustainable means of accessing the station in various ways. These ways included giving free travel mugs plus local cycle, bus and train information to commuters. - Cycle security promotions at train stations, in conjunction with the local police – for example, offering to put security engravings on bicycles. Advice was also given on safety and security more broadly, for example testing whether a bicycle could be adequately seen at night. - Dr Bike sessions at stations and other locations to perform bike checks and repairs/maintenance - Free cycle training, plus ‘bike buddy’ assistance and bike loans. |
| Woking | - Targeted social marketing in selected deprived areas, including setting up a bike club for teenagers in this area and creating a BMX/skate park. - Car Free days, community rides and monthly bike breakfasts (including at railway stations). - Cycle safety ‘lock it or lose it’ campaign in conjunction with the local police. - Lifting a ban on cycling within the (traffic-free) town centre. - Hosting the Tour Series professional cycle ride for one stage, attracting 10,000-15,000 of spectators and involving over 500 riders. - Creating bike loan schemes in some branches of the local government and other organisations. |
| Bristol | - A community grant fund disbursed over £100,000 funding to existing community projects to reach harder-to-reach target groups. Projects included guided bike rides for new cyclists, cycle clubs for older people, bike maintenance workshops for young people, and tandem cycling initiatives for blind or partially sighted people. - Dr. Bike, adult cycle training, and a fleet of good quality bikes for free rental for one month. - Organised a cycle carnival and festival in 2010 attended by 6000 people over two weeks (including 1000 people in a mass bike ride). Also a Cyclescreen film festival; a pedal-powered stage at the Harbour festival; and record levels of attendance at an established mass participation event ‘Bristol’s Biggest Bike Ride’. These were complemented by a presence at smaller community events, e.g. the Festival of Nature and street parties. |
| Shrewsbury | - Engaged an estimated 5200 people through initiatives including 6 cycling festivals, 14 adult guided rides, 14 ‘cycle to work’ days, 9 infrastructure openings and 12 family guided rides. - The highlight among the festivals was the annual festival ‘Bike Fest’, which includes practical sessions, guided rides, bikes to try out (including adapted bikes for people with disabilities) and cycling-related art and entertainment. The main target group was families with young children. - Guided rides included rides aimed at particular groups, including monthly women’s rides and also ‘match day rides’ to home matches by the local football team. - Measures to increase bicycle availability and skills included adult cycle training, short-term bicycle loans, and increasing the number of bicycles in the pool for the local Council. |
| Stoke-on-Trent | - In 2009 and 2010 a week long Bike Week, with multiple cycling events. This was complemented by 1400 smaller events, ranging from route openings to events around the professional races the Tour Series and the Tour of Britain - ‘Match day’ cycle rides to the local football stadium. These attracted large numbers and high-profile local media coverage, with the local Member of Parliament attending four times. - Partnered with a charity in organising guided cycle project for the over 50s in two retirement villages, with grandchildren encouraged to attend during holidays. Partnered in organising cycling events with LGBT (lesbian, gay, bisexual and transgender) community groups. - Multiple Dr. Bike sessions; events at the local university; and an online campaign. |
| Chester | - One route opening, two events around the professional Tour Series, three cycling festivals and numerous local community events. An estimated 20,000 people engaged directly, and 300,000 engaged in events organized in part by Cycle Chester or where the brand was widely promoted. - Promotion through bus and railway advertisements, through radio promotions, through a website, newsletter and e-bulletin. This was complemented by more offbeat promotional measures, for example reclaimed bicycles were decorated with flower baskets and entered into the ‘City in Bloom’ floral competition. - Established a bike loan scheme and funded a carbon-reduction social enterprise to undertake a feasibility study regarding a ‘recycle your bike’ programme. |
| Southport & Ainsdale | - Family oriented cycling festivals, with parades, competitions, information stands, street theatre and a cycle-themed circus. - TV coverage and large crowds brought to watch the professional Tour Series. These were also used as an opportunity to host inter-school races involving - Adult cycle training and guided cycle rides, including for particular target groups such as women only or for the over 50s. The school-based family rides attracted the largest numbers. - Marketing through a website, flyers, a map and local advertisements. |
| Blackpool | - Major spectator events, including the Tour of Britain, Tour Series and Nocturne Series races. - Cycling development/participation events including a guided ride from Manchester to Blackpool, plus smaller community engagement rides including traffic-free local rides, health referral rides and inclusive ‘Wheels for All’ rides. - Bike Club projects set in the hardest to reach areas, plus engaging the Youth Offending Teams in these areas in bike recycling projects. This resulted in improved cycling facilities a highly deprived area, including a new BMX pumptrack. - Establishment of a small public bike hire scheme, and also using bicycle recycling to provide local people with low-cost bikes. - Website, social media campaign and local advertisements. |

Note: Adapted from Goodman et al (1)

Supplemental Table 3: 1991 local authority areas used to identify participants for the intervention and the matched, unfunded and Cycle Cities of Ambition comparison groups

| **Intervention area** | **1991 local authority** | **Matched comparison** | **1991 local authority** | **Unfunded comparison** | **1991 local authority** | **Cycle Cities of Ambition comparison** | **1991 local authority** |
| --- | --- | --- | --- | --- | --- | --- | --- |
| Aylesbury | Aylesbury Vale | Basingstoke and Deane | Basingstoke and Deane | Bath | Bath | Greater Manchester | Bolton |
| Blackpool, Poulton-le- | Blackpool | Bath and North East | Bath | Birmingham | Birmingham |  | Bury |
| Fylde | Fylde | Somerset |  | Braintree | Braintree |  | Manchester |
| Brighton, Hove | Brighton | Bolton | Bolton | Carlisle | Carlisle |  | Oldham |
|  | Hove | Bournemouth | Bournemouth | Corby | Corby |  | Rochdale |
| Bristol, Stoke Gifford, | Bristol | Bromley | Bromley | Camborne/Redruth | Kerrier |  | Salford |
| Mangotsfield |  | Canterbury | Canterbury | Coventry | Coventry |  | Stockport |
| Cambridge/Milton, | Cambridge | Cardiff | Cardiff | Kendal | South Lakeland |  | Tameside |
| Great Shelford, Histon, | South Cambridgeshire | Chelmsford | Chelmsford | Devon Cycling County | East Devon |  | Trafford |
| Harston, Cottenham, |  | Cheltenham | Cheltenham |  | North Devon |  | Wigan |
| Comberton, Girton, |  | Cheshire East | Macclesfield |  | South Hams | West Yorkshire | Bradford |
| Fulbourn, Sawston, |  |  | Congleton |  | Torridge |  | Calderdale |
| Oakington/ |  |  | Crewe and Nantwich |  | Mid Devon |  | Kirklees |
| Longstanton, |  | Chesterfield | Chesterfield |  | Teignbridge |  | Leeds |
| Waterbeach |  | Chorley | Chorley |  | West Devon |  | Wakefield |
| Leighton Buzzard, | South Bedfordshire | County of | Hereford | Doncaster | Doncaster | Birmingham | Birmingham |
| Linslade |  | Herefordshire | South Herefordshire | Weymouth | Weymouth and | West of England | Bath |
| Chester, Guilden | Chester |  | Leominster |  | Portland |  | Wansdyke |
| Sutton, Mickle |  | Dover | Dover | Harlow/ | Harlow |  | Northavon |
| Trafford |  | East Hampshire | East Hampshire | Sawbridgeworth |  |  | Kingswood |
| Colchester | Colchester | East Northamptonshire | East Northamptonshire | Cheltenham | Cheltenham |  | Bristol |
| Darlington | Darlington | East Riding of | East Yorkshire | Gloucester | Gloucester | Newcastle | Newcastle upon Tyne |
| Derby | Derby | Yorkshire | Borough of Beverley | Halifax | Calderdale | Cambridge | Cambridge |
| Exeter | Exeter |  | East Yorkshire | Harrogate/ | Harrogate | Norwich | Norwich |
| Lancaster, Morecambe | Lancaster |  | Holderness | Knaresborough |  | Oxford | Oxford |
| Southport inc. Ainsdale | Sefton |  | Boothferry | Hartlepool | Hartlepool |  |  |
| Shrewsbury, Bayston | Shrewsbury and) | Huntingdonshire | Huntingdonshire | Hereford | Hereford |  |  |
| Hill | Atcham | Isle of Wight | Medina | Newport | Medina |  |  |
| Southend-on-Sea | Southend-on-Sea |  | South Wight | (Isle of Wight) |  |  |  |
| Stoke-on-Trent | Stoke-on-Trent | Kings Lynn and West | Kings Lynn and West | Ashford | Ashford |  |  |
| Woking/Byfleet, | Woking | Norfolk | Norfolk | Preston | Preston |  |  |
| Chobham |  | Kingston-upon-Thames | Kingston upon Thames | Leeds | Leeds |  |  |
| York | York | Leeds | Leeds | Leicester | Leicester |  |  |
|  |  | Maidstone | Maidstone | Grantham | South Kesteven |  |  |
|  |  | Norwich | Norwich | Liverpool | Liverpool |  |  |
|  |  | Oxford | Oxford | Loughborough | Charnwood |  |  |
|  |  | Plymouth | Plymouth | Luton | Luton |  |  |
|  |  | Poole | Poole | Manchester | Manchester |  |  |
|  |  | Portsmouth | Portsmouth | Bletchley | Milton Keynes |  |  |
|  |  | Preston | Preston | Newport Pagnell |  |  |  |
|  |  | Scarborough | Scarborough | Wolverton/ |  |  |  |
|  |  | Southampton | Southampton | Stony Stratford |  |  |  |
|  |  | South Oxfordshire | South Oxfordshire | Gateshead | Gateshead |  |  |
|  |  | St. Albans | St. Albans | Newcastle upon Tyne | Newcastle upon Tyne |  |  |
|  |  | St. Helens | St. Helens | Scunthorpe | Scunthorpe |  |  |
|  |  | Stockton-on-Tees | Stockton-on-Tees | Weston-Super-Mare | Woodspring |  |  |
|  |  | Sunderland | Sunderland | Kettering | Kettering |  |  |
|  |  | Swansea | Swansea | Blyth | Blyth Valley |  |  |
|  |  | Tameside | Tameside | Nottingham | Nottingham |  |  |
|  |  | Three Rivers | Three Rivers | Oldham | Oldham |  |  |
|  |  | Torbay | Torbay | Oxford | Oxford |  |  |
|  |  | Tunbridge Wells | Tunbridge Wells | Nelson | Pendle |  |  |
|  |  | Vale of White Horse | Vale of White Horse | Portsmouth | Portsmouth |  |  |
|  |  | Walsall | Walsall | Reading | Reading |  |  |
|  |  | Warrington | Warrington | Catterick Garrison | Richmondshire |  |  |
|  |  | West Berkshire | Newbury | Richmond |  |  |  |
|  |  | Wigan | Wigan | Oakham | Rutland |  |  |
|  |  | Windsor and | Windsor and | Uppingham |  |  |  |
|  |  | Maidenhead | Maidenhead | Salford | Salford |  |  |
|  |  | Wirral | Wirral | Sheffield | Sheffield |  |  |
|  |  | Worthing | Worthing | Sittingbourne | Swale |  |  |
|  |  |  |  | Slough | Slough |  |  |
|  |  |  |  | Taunton | Taunton Deane |  |  |
|  |  |  |  | Southampton | Southampton |  |  |
|  |  |  |  | Stafford | Stafford |  |  |
|  |  |  |  | Stockport | Stockport |  |  |
|  |  |  |  | Middlesbrough | Middlesbrough |  |  |
|  |  |  |  | Stockton-on-Tees | Stockton-on-Tees |  |  |
|  |  |  |  | Lowestoft | Waveney |  |  |
|  |  |  |  | Ashton-under-Lyne | Tameside |  |  |
|  |  |  |  | Denton (Tameside) |  |  |  |
|  |  |  |  | Dukinfield |  |  |  |
|  |  |  |  | Hyde |  |  |  |
|  |  |  |  | Stalybridge |  |  |  |
|  |  |  |  | Newport | The Wrekin |  |  |
|  |  |  |  | (Telford and Wrekin) |  |  |  |
|  |  |  |  | Andover | Test Valley |  |  |
|  |  |  |  | Grays | Thurrock |  |  |
|  |  |  |  | Paignton | Torbay |  |  |
|  |  |  |  | Torquay |  |  |  |
|  |  |  |  | Castleford | Wakefield |  |  |
|  |  |  |  | Wallsend | North Tyneside |  |  |
|  |  |  |  | Warrington | Warrington |  |  |
|  |  |  |  | Royal Leamington Spa | Warwick |  |  |
|  |  |  |  | Warwick |  |  |  |
|  |  |  |  | Hemel Hempstead | Dacorum |  |  |
|  |  |  |  | Newbury | Newbury |  |  |
|  |  |  |  | Thatcham |  |  |  |
|  |  |  |  | Leigh | Wigan |  |  |
|  |  |  |  | Trowbridge | West Wiltshire |  |  |
|  |  |  |  | Wallasey | Wirral |  |  |
|  |  |  |  | Wolverhampton | Wolverhampton |  |  |
|  |  |  |  | Worcester | Worcester |  |  |
|  |  |  |  | Worthing | Worthing |  |  |

Supplemental Table 4: Matched comparison group 2001 local authorities

| **Intervention Local Authority** | **Most similar Local Authority** | **2nd most similar Local Authority** | **3rd most similar Local Authority** | **4th most similar Local Authority** | **5th most similar Local Authority** |
| --- | --- | --- | --- | --- | --- |
| Aylesbury Vale | West Berkshire | Huntingdonshire | Basingstoke and Deane | East Hertfordshire | Central Bedfordshire |
| Blackpool | Torbay | Scarborough | Isle of Wight | Conwy | Weymouth and Portland |
| Brighton and Hove | Edinburgh, City of | Bournemouth | Cheltenham | Bristol | Norwich |
| Bristol | Cardiff | Southampton | Cheltenham | Trafford | Leeds |
| Cambridge | Oxford | Southampton | Brighton and Hove | Bristol | Kingston-upon-Thames |
| Central Bedfordshire | East Northamptonshire | Huntingdonshire | Basingstoke and Deane | Cherwell | East Hertfordshire |
| Cheshire West and Chester | Cheshire East | Chorley | Warrington | Rugby | Tewkesbury |
| Colchester | Maidstone | Tunbridge Wells | Chelmsford | Ashford | North Somerset |
| Darlington | Stockton-on-Tees | Dover | Chesterfield | North Tyneside | Doncaster |
| Derby | Bolton | Preston | Walsall | Kirklees | Sheffield |
| Exeter | Lancaster | York | Plymouth | Portsmouth | Leeds |
| Lancaster | Canterbury | Exeter | York | Bath and North East Somerset | Swansea |
| Sefton | Wirral | Swansea | St. Helens | Darlington | Stockport |
| Shropshire | County of Herefordshire | East Riding of Yorks | Kings Lynn and West Norfolk | Hambleton | South Somerset |
| South Cambridgeshire | Vale of White Horse | South Oxfordshire | East Hampshire | West Oxfordshire | Harborough |
| Southend-on-Sea | Worthing | Bromley | Poole | Epping Forest | Shepway |
| Stoke-on-Trent | Tameside | Sunderland | Wigan | Barnsley | Halton |
| Woking | St.Albans | Windsor and Maidenhead | Three Rivers | Hertsmere | Elmbridge |
| York | Bath and North East Somerset | Cheltenham | Canterbury | Lancaster | Warwick |

|  | Intervention area |
| --- | --- |
|  | Comparison area |
|  | Area outside dataset, i.e. Scotland |
|  | Area not needed as more similar areas available |

Note: The three most similar local authorities were included for each intervention area. Most similar local authorities as calculated by the Office for National Statistics 2001 Corresponding Local Authority (2)

Supplemental Table 5: Walking and cycling to work in 2001 for each intervention area

|  | Mode of travel to work in 2001 | | |  |
| --- | --- | --- | --- | --- |
|  | Motor vehicle  N (%) | Cycle  N (%) | Walk  N (%) | Total |
|  |  |  |  |  |
| Aylesbury | 330 (89%) | * | * | 372 |
| Blackpool | 371 (89%) | 12 (3%) | 32 (8%) | 415 |
| Brighton | 316 (85%) | * | * | 372 |
| Bristol | 626 (81%) | 39 (5%) | 106 (14%) | 771 |
| Cambridge | 364 (76%) | 69 (14%) | 45 (9%) | 478 |
| Bedford | 228 (91%) | * | * | 250 |
| Chester | 208 (87%) | * | * | 239 |
| Colchester | 261 (85%) | 14 (5%) | 31 (10%) | 306 |
| Darlington | 207 (87%) | * | * | 239 |
| Derby | 396 (81%) | 34 (7%) | 56 (12%) | 486 |
| Exeter | 151 (75%) | 12 (6%) | 38 (19%) | 201 |
| Lancaster | 225 (83%) | 14 (5%) | 32 (12%) | 271 |
| Sefton | 476 (86%) | 16 (3%) | 59 (11%) | 551 |
| Shrewsbury | 177 (84%) | 10 (5%) | 23 (11%) | 210 |
| Southend | 270 (89%) | * | * | 303 |
| Stoke | 446 (84%) | 15 (3%) | 68 (13%) | 529 |
| Woking | 164 (85%) | * | * | 193 |
| York | 112 (60%) | 31 (17%) | 44 (24%) | 187 |

* Suppressed due to low numbers of participants and the potential for disclosure. .

Source: ONS Longitudinal Study

Supplemental Table 6: The characteristics of the intervention and different comparison groups

|  |  | **Intervention  N=6,373** | **England excl. London comparison N=81,953** | **Unfunded comparison N=27,598** | **CCA comparison N=13,133** |
| --- | --- | --- | --- | --- | --- |
| Gender | Male | 3,299 (51.8%) | 43,914 (53.6%) | 14,705 (53.3%) | 6,852 (52.2%) |
|  | Female | 3,074 (48.2%) | 38,039 (46.4%) | 12,893 (46.7%) | 6,281 (47.8%) |
| Age (years) | 16-29 | 1,347 (21.1%) | 17,361 (21.2%) | 6,086 (22.1%) | 2,982 (22.7%) |
|  | 30-39 | 2,107 (33.1%) | 27,101 (33.1%) | 9,395 (34.0%) | 4,565 (34.8%) |
|  | 40-49 | 2,019 (31.7%) | 25,921 (31.6%) | 8,535 (30.9%) | 3,998 (30.4%) |
|  | 50+ | 900 (14.1%) | 11,570 (14.1%) | 3,582 (13.0%) | 1,588 (12.1%) |
| Ethnicity | Minority ethnicity | 241 ( 3.8%) | 3,897 ( 4.8%) | 1,960 ( 7.1%) | 1,195 ( 9.1%) |
|  | White | 6,132 (96.2%) | 78,056 (95.2%) | 25,638 (92.9%) | 11,938 (90.9%) |
| Highest qualification | Less than 5 GCSE A-C | 2,520 (39.5%) | 33,720 (41.1%) | 11,495 (41.7%) | 5,438 (41.4%) |
|  | 5 GCSE A-C no degree | 2,308 (36.2%) | 31,509 (38.4%) | 10,457 (37.9%) | 4,795 (36.5%) |
|  | Degree | 1,545 (24.2%) | 16,724 (20.4%) | 5,646 (20.5%) | 2,900 (22.1%) |
| Marital Status | Unmarried | 2,806 (44.0%) | 32,914 (40.2%) | 11,715 (42.4%) | 5,680 (43.2%) |
|  | Married | 3,567 (56.0%) | 49,039 (59.8%) | 15,883 (57.6%) | 7,453 (56.8%) |
| Limiting long term illness | No illness | 6,029 (94.6%) | 77,770 (94.9%) | 26,229 (95.0%) | 12,457 (94.9%) |
|  | Has illness | 344 ( 5.4%) | 4,183 ( 5.1%) | 1,369 ( 5.0%) | 676 ( 5.1%) |
| Self-reported health | Good | 5,059 (79.4%) | 64,575 (78.8%) | 21,572 (78.2%) | 10,212 (77.8%) |
|  | Fairly good | 1,161 (18.2%) | 15,514 (18.9%) | 5,371 (19.5%) | 2,599 (19.8%) |
|  | Not good | 153 ( 2.4%) | 1,864 ( 2.3%) | 655 ( 2.4%) | 322 ( 2.5%) |
| Working status | Full time | 4,923 (77.2%) | 63,429 (77.4%) | 21,419 (77.6%) | 10,170 (77.4%) |
|  | Part time | 1,450 (22.8%) | 18,524 (22.6%) | 6,179 (22.4%) | 2,963 (22.6%) |
| Number of cars/vans | No car | 571 ( 9.0%) | 5,591 ( 6.8%) | 2,503 ( 9.1%) | 1,355 (10.3%) |
|  | One car | 2,691 (42.2%) | 32,199 (39.3%) | 11,773 (42.7%) | 5,820 (44.3%) |
|  | More than one car | 3,111 (48.8%) | 44,163 (53.9%) | 13,322 (48.3%) | 5,958 (45.4%) |
| Housing tenure | Owner | 5,347 (83.9%) | 69,862 (85.2%) | 23,117 (83.8%) | 11,117 (84.6%) |
|  | Non-owner | 1,026 (16.1%) | 12,091 (14.8%) | 4,481 (16.2%) | 2,016 (15.4%) |
| Occupation group | Managerial | 2,480 (38.9%) | 30,284 (37.0%) | 9,884 (35.8%) | 4,821 (36.7%) |
|  | Intermediate | 2,119 (33.2%) | 27,375 (33.4%) | 9,195 (33.3%) | 4,357 (33.2%) |
|  | (Semi-)routine | 1,774 (27.8%) | 24,294 (29.6%) | 8,519 (30.9%) | 3,955 (30.1%) |
| Commute mode in 2001 | Motor vehicle | 5,328 (83.6%) | 71,217 (86.9%) | 23,849 (86.4%) | 11,511 (87.6%) |
|  | Cycle | 306 ( 4.8%) | 2,676 ( 3.3%) | 838 ( 3.0%) | 278 ( 2.1%) |
|  | Walk | 739 (11.6%) | 8,060 ( 9.8%) | 2,911 (10.5%) | 1,344 (10.2%) |
| Area-level deprivation | Least deprived | 1,354 (21.3%) | 19,051 (23.3%) | 4,021 (14.6%) | 998 ( 7.6%) |
|  | 2 | 1,086 (17.1%) | 18,657 (22.8%) | 4,875 (17.7%) | 2,633 (20.1%) |
|  | 3 | 1,662 (26.1%) | 17,327 (21.2%) | 6,370 (23.1%) | 2,852 (21.8%) |
|  | 4 | 1,467 (23.0%) | 15,537 (19.0%) | 6,502 (23.6%) | 3,326 (25.4%) |
|  | Most deprived | 798 (12.5%) | 11,224 (13.7%) | 5,771 (21.0%) | 3,292 (25.1%) |
| Moved home between 2001 and 2011 | Did not move home | 4,150 (65.1%) | 54,308 (66.3%) | 17,852 (64.7%) | 8,596 (65.5%) |
|  | Moved home | 2,223 (34.9%) | 27,645 (33.7%) | 9,746 (35.3%) | 4,537 (34.5%) |
| Dependent child in household | No dependent child(ren) | 3,313 (52.1%) | 40,065 (48.9%) | 13,575 (49.2%) | 6,303 (48.0%) |
|  | 1+ dependent child(ren) | 3,051 (47.9%) | 41,820 (51.1%) | 13,999 (50.8%) | 6,824 (52.0%) |

Data are presented as n (%). GCSE – General Certificate of Secondary Education
Source: ONS Longitudinal Study

Supplemental Table 7: DID estimates across the three outcomes and four comparison groups in the unstratified and stratified analyses

|  | **Cycle** | **Walk** | **Walk or cycle** |
| --- | --- | --- | --- |
|  | **OR (95% CI)** | **OR (95% CI)** | **OR (95% CI)** |
| **Unstratified** |  |  |  |
| Matched | 1.08 (1.26 to 0.92) | 1.18 (1.32 to 1.06) | 1.16 (1.27 to 1.06) |
| All non-London | 1.21 (1.38 to 1.05) | 1.11 (1.22 to 1.01) | 1.14 (1.24 to 1.05) |
| CCA | 0.96 (1.16 to 0.79) | 1.18 (1.33 to 1.05) | 1.13 (1.25 to 1.02) |
| Unfunded | 1.07 (1.25 to 0.92) | 1.14 (1.27 to 1.02) | 1.13 (1.23 to 1.03) |
|  |  |  |  |
| **Men** |  |  |  |
| Matched | 0.91 (1.10 to 0.76) | 1.08 (1.30 to 0.90) | 1.00 (1.14 to 0.87) |
| All non-London | 1.05 (1.24 to 0.89) | 1.04 (1.21 to 0.88) | 1.03 (1.16 to 0.92) |
| CCA | 0.84 (1.04 to 0.67) | 1.10 (1.34 to 0.91) | 0.98 (1.14 to 0.84) |
| Unfunded | 0.95 (1.14 to 0.79) | 1.03 (1.23 to 0.87) | 0.99 (1.12 to 0.87) |
|  |  |  |  |
| **Women** |  |  |  |
| Matched | 1.56 (2.10 to 1.16) | 1.24 (1.43 to 1.08) | 1.32 (1.51 to 1.16) |
| All non-London | 1.61 (2.08 to 1.25) | 1.16 (1.31 to 1.02) | 1.25 (1.40 to 1.11) |
| CCA | 1.25 (1.84 to 0.84) | 1.23 (1.43 to 1.06) | 1.29 (1.48 to 1.12) |
| Unfunded | 1.36 (1.82 to 1.01) | 1.21 (1.38 to 1.06) | 1.27 (1.44 to 1.12) |
|  |  |  |  |
| **16-39 years** |  |  |  |
| Matched | 0.97 (1.20 to 0.78) | 1.19 (1.38 to 1.02) | 1.12 (1.28 to 0.99) |
| All non-London | 1.15 (1.38 to 0.95) | 1.09 (1.26 to 0.95) | 1.12 (1.25 to 0.99) |
| CCA | 0.95 (1.22 to 0.73) | 1.21 (1.43 to 1.03) | 1.15 (1.32 to 1.00) |
| Unfunded | 1.02 (1.26 to 0.83) | 1.13 (1.31 to 0.98) | 1.11 (1.25 to 0.98) |
|  |  |  |  |
| **40+ years** |  |  |  |
| Matched | 1.27 (1.61 to 1.01) | 1.18 (1.38 to 1.01) | 1.21 (1.39 to 1.06) |
| All non-London | 1.29 (1.58 to 1.06) | 1.14 (1.31 to 0.99) | 1.18 (1.33 to 1.05) |
| CCA | 0.98 (1.30 to 0.74) | 1.15 (1.35 to 0.97) | 1.11 (1.28 to 0.96) |
| Unfunded | 1.16 (1.46 to 0.92) | 1.16 (1.34 to 1.00) | 1.16 (1.32 to 1.02) |
|  |  |  |  |
| **No degree** |  |  |  |
| Matched | 1.03 (1.25 to 0.84) | 1.16 (1.31 to 1.03) | 1.13 (1.26 to 1.02) |
| All non-London | 1.13 (1.35 to 0.95) | 1.08 (1.21 to 0.97) | 1.10 (1.21 to 1.00) |
| CCA | 0.92 (1.17 to 0.72) | 1.17 (1.33 to 1.02) | 1.11 (1.24 to 0.99) |
| Unfunded | 1.02 (1.23 to 0.84) | 1.11 (1.25 to 0.99) | 1.09 (1.21 to 0.98) |
|  |  |  |  |
| **Degree** |  |  |  |
| Matched | 1.10 (1.45 to 0.83) | 1.19 (1.53 to 0.93) | 1.18 (1.44 to 0.97) |
| All non-London | 1.21 (1.54 to 0.95) | 1.14 (1.42 to 0.92) | 1.20 (1.42 to 1.01) |
| CCA | 1.01 (1.40 to 0.73) | 1.14 (1.50 to 0.86) | 1.11 (1.38 to 0.89) |
| Unfunded | 1.08 (1.42 to 0.82) | 1.17 (1.48 to 0.92) | 1.17 (1.41 to 0.97) |
|  |  |  |  |
| **Low deprivation** |  |  |  |
| Matched | 0.91 (1.19 to 0.69) | 1.15 (1.37 to 0.97) | 1.09 (1.26 to 0.93) |
| All non-London | 1.12 (1.41 to 0.89) | 1.11 (1.29 to 0.95) | 1.11 (1.27 to 0.97) |
| CCA | 0.80 (1.08 to 0.59) | 1.12 (1.33 to 0.94) | 1.05 (1.22 to 0.90) |
| Unfunded | 0.93 (1.19 to 0.72) | 1.15 (1.35 to 0.98) | 1.09 (1.25 to 0.95) |
|  |  |  |  |
| **High deprivation** |  |  |  |
| Matched | 1.06 (1.37 to 0.83) | 1.10 (1.33 to 0.91) | 1.09 (1.27 to 0.94) |
| All non-London | 1.18 (1.47 to 0.94) | 1.07 (1.27 to 0.90) | 1.10 (1.27 to 0.96) |
| CCA | 0.93 (1.28 to 0.67) | 1.08 (1.35 to 0.86) | 1.03 (1.24 to 0.86) |
| Unfunded | 1.08 (1.39 to 0.83) | 1.05 (1.27 to 0.87) | 1.05 (1.23 to 0.90) |
|  | |  |  |

Source: ONS Longitudinal Study

Estimates adjusted for: age, gender, ethnicity, highest educational qualification, occupation based socio-economic group, long-term illness, self-reported health, number of cars, housing tenure, working status, marital status and quintile of Carstairs index of ward of residence (a composite measure of area-level deprivation.

Comparison groups: Matched = comparison group matched using Office for National Statistics Corresponding Authorities; All non-London = all participant resident in England outside of Greater London; CCA = Areas successful in apply for funding through the Cycling Cities of Ambition funding scheme; Reject: Areas that applied for CDT or CCT funding but were unsuccessful.

OR = Odd ratio; DID = Difference-in-difference

Supplemental Table 8: Association between living in an intervention area and taking up cycling and maintaining cycling in 2011 compared with the matched comparison group

|  | **Taking up cycling** | | | | **Maintaining cycling** | | | |
| --- | --- | --- | --- | --- | --- | --- | --- | --- |
|  | **Unadjusted** | | **Adjusted** | | **Unadjusted** | | **Adjusted** | |
|  | **OR (95% CI)** | **P-value** | **OR (95% CI)** | **P-value** | **OR (95% CI)** | **P-value** | **OR (95% CI)** | **P-value** |
|  |  |  |  |  |  |  |  |  |
| Pooled | 1.49 (1.24, 1.80) | <0.001 | 1.47 (1.21, 1.77) | <0.001 | 1.26 (0.96, 1.66) | 0.101 | 1.21 (0.91, 1.61) | 0.199 |
|  |  |  |  |  |  |  |  |  |
| Male | 1.23 (0.97, 1.57) | 0.083 | 1.19 (0.93, 1.51) | 0.164 | 1.15 (0.84, 1.59) | 0.382 | 1.06 (0.76, 1.50) | 0.722 |
| Female | 2.20 (1.62, 3.00) | <0.001 | 2.13 (1.56, 2.91) | <0.001 | 1.63 (0.94, 2.81) | 0.080 | 1.65 (0.91, 2.98) | 0.099 |
|  |  |  |  |  |  |  |  |  |
| Aged 16-39 years | 1.31 (1.04, 1.66) | 0.021 | 1.27 (1.01, 1.61) | 0.044 | 1.30 (0.90, 1.87) | 0.166 | 1.24 (0.85, 1.83) | 0.268 |
| Aged 40+ years | 1.97 (1.44, 2.71) | <0.001 | 1.95 (1.42, 2.69) | <0.001 | 1.23 (0.81, 1.89) | 0.330 | 1.13 (0.72, 1.78) | 0.582 |
|  |  |  |  |  |  |  |  |  |
| No degree | 1.33 (1.06, 1.66) | 0.014 | 1.31 (1.04, 1.64) | 0.023 | 1.05 (0.75, 1.46) | 0.777 | 1.07 (0.76, 1.51) | 0.713 |
| Degree | 1.93 (1.39, 2.70) | <0.001 | 1.84 (1.31, 2.59) | <0.001 | 1.64 (0.97, 2.78) | 0.064 | 1.81 (1.01, 3.26) | 0.046 |
|  |  |  |  |  |  |  |  |  |
| Low area deprivation | 1.33 (0.98, 1.80) | 0.067 | 1.34 (0.98, 1.82) | 0.065 | 1.20 (0.76, 1.90) | 0.437 | 1.17 (0.71, 1.92) | 0.546 |
| High area deprivation | 1.42 (1.04, 1.94) | 0.026 | 1.42 (1.04, 1.95) | 0.027 | 1.27 (0.81, 1.98) | 0.299 | 1.26 (0.78, 2.01) | 0.345 |

Estimates adjusted for: age, gender, ethnicity, highest educational qualification, occupation based socio-economic group, long-term illness, self-reported health, number of cars, housing tenure, working status, marital status and quintile of Carstairs index of ward of residence (a composite measure of area-level deprivation.

OR – Odds ratio

Source: ONS Longitudinal Study

Supplemental Table 9: DID effect estimates for sensitivity analyses for each outcome and strata

|  | **Cycle** | **Walk** | **Walk or cycle** |
| --- | --- | --- | --- |
|  | **OR (95% CI)** | **OR (95% CI)** | **OR (95% CI)** |
| **Unstratified** |  |  |  |
| Matched | 1.08 (1.26 to 0.92) | 1.18 (1.32 to 1.06) | 1.16 (1.27 to 1.06) |
| Excl. Cambridge | 1.11 (1.32 to 0.93) | 1.19 (1.33 to 1.07) | 1.18 (1.30 to 1.07) |
| Excl. imputed | 0.99 (1.17 to 0.83) | 1.19 (1.34 to 1.06) | 1.14 (1.26 to 1.03) |
| Excl. rural | 1.05 (1.25 to 0.88) | 1.20 (1.36 to 1.07) | 1.17 (1.30 to 1.06) |
| Adj. child | 1.07 (1.26 to 0.91) | 1.19 (1.33 to 1.06) | 1.16 (1.28 to 1.06) |
| Adj. move home | 1.08 (1.26 to 0.92) | 1.18 (1.32 to 1.06) | 1.16 (1.27 to 1.06) |
|  |  |  |  |
| **Men** |  |  |  |
| Matched | 0.91 (1.10 to 0.76) | 1.08 (1.30 to 0.90) | 1.00 (1.14 to 0.87) |
| Excl. Cambridge | 0.93 (1.14 to 0.76) | 1.08 (1.30 to 0.90) | 1.01 (1.16 to 0.88) |
| Excl. imputed | 0.86 (1.05 to 0.70) | 1.11 (1.34 to 0.91) | 0.98 (1.13 to 0.85) |
| Excl. rural | 0.86 (1.05 to 0.70) | 1.11 (1.35 to 0.91) | 0.98 (1.14 to 0.85) |
| Adj. child | 0.91 (1.10 to 0.75) | 1.10 (1.32 to 0.92) | 1.00 (1.15 to 0.88) |
| Adj. move home | 0.91 (1.10 to 0.76) | 1.08 (1.30 to 0.90) | 1.00 (1.14 to 0.87) |
|  |  |  |  |
| **Women** |  |  |  |
| Matched | 1.56 (2.10 to 1.16) | 1.24 (1.43 to 1.08) | 1.32 (1.51 to 1.16) |
| Excl. Cambridge | 1.69 (2.38 to 1.21) | 1.27 (1.46 to 1.10) | 1.36 (1.55 to 1.19) |
| Excl. imputed | 1.32 (1.83 to 0.95) | 1.25 (1.46 to 1.08) | 1.30 (1.49 to 1.13) |
| Excl. rural | 1.72 (2.41 to 1.23) | 1.26 (1.47 to 1.09) | 1.37 (1.57 to 1.19) |
| Adj. child | 1.54 (2.08 to 1.15) | 1.24 (1.43 to 1.08) | 1.32 (1.51 to 1.16) |
| Adj. move home | 1.56 (2.10 to 1.16) | 1.24 (1.43 to 1.08) | 1.32 (1.51 to 1.16) |
|  |  |  |  |
| **16-39 years** |  |  |  |
| Matched | 0.97 (1.20 to 0.78) | 1.19 (1.38 to 1.02) | 1.12 (1.28 to 0.99) |
| Excl. Cambridge | 0.95 (1.20 to 0.76) | 1.22 (1.43 to 1.04) | 1.14 (1.31 to 1.00) |
| Excl. imputed | 0.88 (1.11 to 0.70) | 1.19 (1.41 to 1.01) | 1.09 (1.25 to 0.95) |
| Excl. rural | 0.93 (1.17 to 0.74) | 1.18 (1.39 to 1.00) | 1.10 (1.27 to 0.96) |
| Adj. child | 0.96 (1.19 to 0.77) | 1.20 (1.40 to 1.02) | 1.13 (1.28 to 0.99) |
| Adj. move home | 0.97 (1.20 to 0.78) | 1.19 (1.38 to 1.02) | 1.12 (1.28 to 0.99) |
|  |  |  |  |
| **40+ years** |  |  |  |
| Matched | 1.27 (1.61 to 1.01) | 1.18 (1.38 to 1.01) | 1.21 (1.39 to 1.06) |
| Excl. Cambridge | 1.41 (1.83 to 1.09) | 1.17 (1.37 to 1.00) | 1.24 (1.42 to 1.08) |
| Excl. imputed | 1.18 (1.52 to 0.92) | 1.21 (1.43 to 1.03) | 1.21 (1.39 to 1.05) |
| Excl. rural | 1.27 (1.66 to 0.97) | 1.25 (1.48 to 1.05) | 1.27 (1.47 to 1.09) |
| Adj. child | 1.27 (1.61 to 1.01) | 1.19 (1.38 to 1.02) | 1.22 (1.39 to 1.07) |
| Adj. move home | 1.27 (1.61 to 1.01) | 1.18 (1.38 to 1.01) | 1.21 (1.39 to 1.06) |
|  |  |  |  |
| **No degree** |  |  |  |
| Matched | 1.03 (1.25 to 0.84) | 1.16 (1.31 to 1.03) | 1.13 (1.26 to 1.02) |
| Excl. Cambridge | 0.98 (1.21 to 0.80) | 1.17 (1.33 to 1.04) | 1.13 (1.26 to 1.01) |
| Excl. imputed | 0.94 (1.16 to 0.75) | 1.19 (1.36 to 1.04) | 1.12 (1.26 to 1.00) |
| Excl. rural | 1.01 (1.25 to 0.81) | 1.18 (1.35 to 1.03) | 1.14 (1.28 to 1.02) |
| Adj. child | 1.02 (1.24 to 0.84) | 1.16 (1.31 to 1.02) | 1.13 (1.25 to 1.01) |
| Adj. move home | 1.03 (1.25 to 0.84) | 1.16 (1.31 to 1.03) | 1.13 (1.26 to 1.02) |
|  |  |  |  |
| **Degree** |  |  |  |
| Matched | 1.10 (1.45 to 0.83) | 1.19 (1.53 to 0.93) | 1.18 (1.44 to 0.97) |
| Excl. Cambridge | 1.35 (1.88 to 0.97) | 1.23 (1.60 to 0.94) | 1.32 (1.63 to 1.06) |
| Excl. imputed | 1.00 (1.34 to 0.74) | 1.16 (1.51 to 0.89) | 1.12 (1.37 to 0.91) |
| Excl. rural | 1.05 (1.43 to 0.77) | 1.25 (1.64 to 0.95) | 1.20 (1.49 to 0.97) |
| Adj. child | 1.09 (1.44 to 0.82) | 1.23 (1.59 to 0.96) | 1.21 (1.47 to 0.99) |
| Adj. move home | 1.10 (1.45 to 0.83) | 1.19 (1.53 to 0.93) | 1.18 (1.44 to 0.97) |
|  |  |  |  |
| **Low deprivation** |  |  |  |
| Matched | 0.91 (1.19 to 0.69) | 1.15 (1.37 to 0.97) | 1.09 (1.26 to 0.93) |
| Excl. Cambridge | 0.94 (1.25 to 0.70) | 1.14 (1.36 to 0.96) | 1.09 (1.28 to 0.94) |
| Excl. imputed | 0.86 (1.16 to 0.64) | 1.23 (1.49 to 1.02) | 1.13 (1.33 to 0.96) |
| Excl. rural | 0.92 (1.21 to 0.70) | 1.15 (1.37 to 0.96) | 1.09 (1.27 to 0.93) |
| Adj. child | 0.91 (1.19 to 0.69) | 1.16 (1.38 to 0.97) | 1.09 (1.27 to 0.94) |
| Adj. move home | 0.91 (1.19 to 0.69) | 1.15 (1.37 to 0.97) | 1.09 (1.26 to 0.93) |
|  |  |  |  |
| **High deprivation** |  |  |  |
| Matched | 1.06 (1.37 to 0.83) | 1.10 (1.33 to 0.91) | 1.09 (1.27 to 0.94) |
| Excl. Cambridge | 1.05 (1.39 to 0.80) | 1.15 (1.39 to 0.95) | 1.12 (1.32 to 0.96) |
| Excl. imputed | 0.99 (1.31 to 0.75) | 1.04 (1.26 to 0.85) | 1.02 (1.20 to 0.87) |
| Excl. rural | 1.05 (1.45 to 0.76) | 1.14 (1.44 to 0.90) | 1.12 (1.35 to 0.92) |
| Adj. child | 1.05 (1.36 to 0.82) | 1.12 (1.35 to 0.92) | 1.10 (1.28 to 0.94) |
| Adj. move home | 1.06 (1.37 to 0.83) | 1.10 (1.33 to 0.91) | 1.09 (1.27 to 0.94) |

Estimates adjusted for: age, gender, ethnicity, highest educational qualification, occupation based socio-economic group, long-term illness, self-reported health, number of cars, housing tenure, working status, marital status and quintile of Carstairs index of ward of residence (a composite measure of area-level deprivation.

OR = Odd ratio

Comparisons: Matched = comparison group matched using Office for National Statistics Corresponding Authorities (main analyses); Excl. Cambridge = main analyses excluding Cambridge and its matched control areas; Excl. imputed = main analyses excluding participants with imputed values for any variables of interest; Excl. rural = main analyses excluding those living in areas with a population density < 1000 people per square kilometre; Adj. child = additional adjustment for the presence of at least one dependent child in the household; Adj. move home = additional adjustment for moving home within the study period.

Source: ONS Longitudinal Study

# References

1. Goodman A, Panter J, Sharp SJ, Ogilvie D. Effectiveness and equity impacts of town-wide cycling initiatives in England: A longitudinal, controlled natural experimental study. Soc Sci Med. 2013 Nov 1;97:228–37.

2. Office for National Statistics. Corresponding local authorities [Internet]. 2001 [cited 2022 Jul 4]. Available from: https://webarchive.nationalarchives.gov.uk/ukgwa/20160128205836/http:/www.ons.gov.uk/ons/guide-method/geography/products/area-classifications/ns-area-classifications/ns-2001-area-classifications/corresponding-authorities/local-authorities/index.html
